# *PLoS Medicine's* Advisory Group on Publication Ethics

**DOI:** 10.1371/journal.pmed.0040081

**Published:** 2007-02-27

**Authors:** The PLoS Medicine Editors

## Abstract

*PLoS Medicine's* external advisory committee is now two years old. What has the committee achieved and how has it helped to shape policies at the journal?

In March 2005, we announced the appointment of an external advisory committee “to advise us on individual cases for which we are concerned about competing interests or broader ethical questions” [1]. Two years on, what has the committee achieved and how has it helped to shape policies at the journal?

The committee is a “virtual” one, with seven members in two countries—the United States and South Africa. When the *PLoS Medicine* editors have a case that presents us with an ethical dilemma, we submit anonymized details to the committee and ask for its advice. Committee members were chosen for their very broad expertise, not just in medical ethics but also in law, health policy, medical journalism and editing, clinical medicine, and clinical research. In response to our request for advice, the committee members share their opinions via a listserv, and then the committee chair presents a summary of the committee's advice to the editors.

Since the committee was appointed, it has responded to ten queries. These queries can be divided into three broad themes: (1) competing interests; (2) preserving patient anonymity; and (3) the borderline between a research paper and a programmatic description.

## Competing Interests

From its very first issue, *PLoS Medicine* asked all authors to declare any competing interests (http://journals.plos.org/plosmedicine/competing.php), which we placed prominently alongside the authors' biographical information. Initially we allowed authors to acknowledge their funding sources at the end of the article. This policy meant that some authors would declare that “no competing interests exist,” and yet in the funding statement at the end of their article would state that they had received funding from pharmaceutical companies.

We presented examples of such cases to the committee, which was unanimous in believing that authors are poor judges of what constitutes a competing interest and that we should revise our policy. We therefore implemented the committee's recommendations to: (1) make sure that competing interests and funding sources were declared together at the beginning of every article, and (2) ask authors to state what role the funder played in the submission and preparation of the article. Our policy regarding articles submitted spontaneously (rather than commissioned) for the Magazine section goes even further. If authors proposing a topic have competing interests that could reasonably be perceived as affecting their ability to write an objective Magazine piece, we will decline their article.

We were also concerned that some of our research papers had the potential to be “spun” favorably by drug companies, since these papers showed the benefit of a particular blockbuster drug. In all cases, we as editors—together with the academic editor and independent peer reviewers—were convinced that the studies were rigorous and should be published, but we wondered, “What can we do to reduce the risk of the results being ‘spun’?”

The committee's overall opinion was reflected by one member, who wrote: “If you think the article is sound enough to publish, you should publish it. You can't control the spin, but you can make sure that full information (on author connections, financial ties, etc) is available in the press release. Hopefully, this will prompt journalists to ask relevant questions.”

In response to this advice, we have—where appropriate—added information about authors' competing interests within the press release. Unfortunately, research on media reports about blockbuster drugs has shown that journalists do poorly at reporting authors' competing interests [2,3]. And so even if we add these interests to our press releases we recognize that journalists may not report them, but we will continue to encourage transparency as best as we can.

## Preserving Patient Anonymity

*PLoS Medicine* adheres to the principle that a case report that arises from the confidential doctor–patient relationship cannot be published unless the patient (or family) has given written consent. But sometimes authors say that they are simply unable to obtain consent (for example, the patient has moved to a different country) and they offer to anonymize the report. If there are educational benefits in publishing such a report, and these benefits outweigh the risks of harm (for example, patients identify themselves and feel that their privacy has been violated), would it be ethically acceptable to publish the report without patient consent?

This question arose when we were approached by a legal organization that has amassed a wealth of illuminating medico-legal cases that could potentially educate doctors on how to stay out of court. Many of the cases involve patients who were harmed or died as a result of medical errors. We asked the organization if it had obtained consent from the patient or relatives to publish case details. It had not—but the organization promised to anonymize all patient details. “We feel the benefits of educating doctors about medical error outweigh any ethical concerns about the invasion of an individual's privacy,” wrote a representative of the organization. “We take great care to anonymise the cases in such a way that we feel it is extremely unlikely that anyone could be identified by the narrative.”

Our advisory committee was unanimous in believing that anonymization would not be sufficient to protect patient anonymity. As one committee member said, we could never be sure that the organization had done a thorough enough job of the anonymization process, and therefore, “we should assume there is a chance that the patient's privacy will be violated,” with a risk of causing harm. As a result of the committee's advice, we declined to publish these medico-legal cases.

Several of the questions that we put to the committee were related to the ethics of publishing “stock” photos of patients (these usually come from news agencies) in Magazine articles when we did not have the patient's written consent. Most medical journals get around this problem by adopting a position of “assumed consent.” The *BMJ*, for example, justifies publishing such photos as follows: “We believe that the *BMJ* would be at a disadvantage among other media if we didn't use such images, and pictures can often tell a story more powerfully than words. But we cannot take responsibility for the consent of people who are shown in pictures that we have obtained from agencies, libraries, other publications, and other commercial sources…we assume that they and their photographers have obtained relevant permission from models in any images showing people” [4]. We asked our committee: should *PLoS Medicine* also assume consent to use photos of patients from news agencies in our Magazine articles?

The committee's deliberations centered on a crucial difference between the *BMJ* and *PLoS Medicine*. Unlike the *BMJ*, we publish all materials under the Creative Commons Attribution License (http://journals.plos.org/plosmedicine/license.php), which allows anyone to reuse the materials for any legal purpose. While the patients in the photos might have given consent to the news agency for their photo to be taken (and this is only an assumption), they certainly did not consent to the Creative Commons licensing agreement. And so the committee felt strongly that we should not use such photos, a position we have adopted.

## Is a Program Description a Research Paper?

The most recent—and most contentious—ethical dilemma that we have faced concerned a paper that fell in the grey area between research and a programmatic description. The authors wished to describe their experience of delivering an innovative health care program under extremely difficult conditions. The authors made no mention of Institutional Review Board (IRB) approval, and so we asked the committee whether it considered this sort of paper as a “study” that required prior IRB approval.

The committee was divided on this question. Some committee members felt that describing a health intervention in order to educate readers does not constitute research and so does not need IRB approval. “We don't hold educational programs to this standard, nor do we hold government programs,” wrote one committee member, adding that “this seems to be to be a serious case of IRB creep” and suggesting that we were on a slippery slope to requiring “IRB approval even to publish a poem.”

But other members felt strongly that as soon as an author is using patient data, there is a requirement for IRB approval. One member wrote that publishing a program description is “analogous to secondary data analyses—where data are collected from individuals for one purpose, but then used by researchers for another. In this case, it is still necessary to safeguard the individuals who provide the data.”

Given the array of differing opinions from the committee, in the first instance we asked for some additional clarifications from the authors as to: (1) whether they sought IRB approval; (2) if they did not seek such approval, what was their rationale; and (3) whether in fact an IRB had confirmed that no approval was necessary for a programmatic description.

The authors explained that they had not sought prior IRB approval because they considered the paper to be “purely descriptive: no hypotheses have been tested; no specific data collection or analyses have been performed for research purposes, nor have any interviews or surveys been undertaken.” All the data in their paper, they said, “are derived from routine programme reporting and come from patient files completed by the doctor at the time of consultation.”

In response to our queries, the authors also asked their IRB to consider the question of whether this paper constituted research and, as such, whether it required post hoc ethical approval. The IRB wrote: “The contents of this paper are retrospective and descriptive and do not qualify as research as per the commonly understood meaning of this term.”

We were satisfied by these clarifications from the authors and their IRB, and we plan to publish the paper. We have asked the authors to include a discussion of these ethical issues within the paper itself.

## Conclusion

We believe that the external advisory committee has provided a valuable service, guiding, educating, and helping us to set policies that we hope have made the journal more “ethically robust.” We have particularly valued the fact that the different committee members bring such a diversity of views, and that the committee is able to give very rapid advice. We would like to thank the committee members, who are listed in Table S2 of reference [1], and we look forward to our ongoing collaboration.
